# Vacuolar H^+^-ATPase Subunit V0C Regulates Aerobic Glycolysis of Esophageal Cancer Cells via PKM2 Signaling

**DOI:** 10.3390/cells8101137

**Published:** 2019-09-24

**Authors:** Sung Wook Son, Gia Cac Chau, Seong-Tae Kim, Sung Hee Um

**Affiliations:** 1 Department of Molecular Cell Biology, Samsung Biomedical Research Institute, Sungkyunkwan University School of Medicine, Suwon, Gyeonggi-do 16419, Korea; ssw4069@gmail.com (S.W.S.); chaugiacac@gmail.com (G.C.C.); stkim@skku.edu (S.-T.K.); 2 Department of Health Sciences and Technology, Samsung Advanced Institute for Health Sciences and Technology, Samsung Medical Center, Sungkyunkwan University, Seoul 06351, Korea; 3 Biomedical Institute Convergence at Sungkyunkwan University, Suwon, Gyeonggi-do 16419, Korea

**Keywords:** vacuolar H^+^-ATPase subunit V0C, aerobic glycolysis, esophageal cancer cell, pyruvate kinase muscle isozyme 2 (PKM2), motility

## Abstract

The vacuolar H^+^-adenosine triphosphatase (ATPase) subunit V0C (ATP6V0C), a proton-conducting, pore-forming subunit of vacuolar ATPase, maintains pH homeostasis and induces organelle acidification. The intracellular and extracellular pH of cancer cells affects their growth; however, the role of ATP6V0C in highly invasive esophageal cancer cells (ECCs) remains unclear. In this study, we examined the role of ATP6V0C in glucose metabolism in ECCs. The ATP6V0C depletion attenuated ECC proliferation, invasion, and suppressed glucose metabolism, as indicated by reduced glucose uptake and decreased lactate and adenosine triphosphate (ATP) production in cells. Consistent with this, expression of glycolytic enzyme and the extracellular acidification rate (ECAR) were also decreased by ATP6V0C knockdown. Mechanistically, ATP6V0C interacted with pyruvate kinase isoform M2 (PKM2), a key regulator of glycolysis in ECCs. The ATP6V0C depletion reduced PKM2 phosphorylation at tyrosine residue 105 (Tyr^105^), leading to inhibition of nuclear translocation of PKM2. In addition, ATP6V0C was recruited at hypoxia response element (HRE) sites in the lactate dehydrogenase A (*LDHA*) gene for glycolysis. Thus, our data suggest that ATP6V0C enhances aerobic glycolysis and motility in ECCs.

## 1. Introduction

Esophageal cancer is one of the most lethal malignancies in the world [1]. In the majority of cases, esophageal cancer is a squamous cell carcinoma and carries a poor prognosis due to the late diagnosis after metastasis [1]. Despite recent advances in diagnosis and treatment using chemotherapy or radiation, the five-year survival rate in esophageal squamous cell carcinoma (ESCC) patients is low (up to 30–45%) because of high mortality [2].

Cancer cells constitutively activate glucose metabolism to promote cell survival and proliferation [3]. Through aerobic glycolysis, cancer cells generate energy, such as adenosine triphosphate (ATP), even in the presence of abundant oxygen [4]. Although aerobic glycolysis is inefficient in generating ATP, cancer cells compensate for this inefficiency by increasing glucose uptake and lactate production [5]. Consistent with this result, positron emission tomography scans with 2-deoxy-2-[^18^F] fluoro-D-glucose (FDG) have revealed excessive glucose accumulation within human esophageal tumors [6], indicating that esophageal cancer cells (ECCs) are highly active in glycolysis. However, how glucose metabolism is modified in highly aggressive ECCs is unclear.

Glucose and amino acids are critical nutrients for cancer cell metabolism and growth [7,8]. Glucose-rich extracellular environments increase glucose uptake and glucose transporter 1 (Glut 1) expression through the mammalian target of rapamycin (mTOR) [9]. Amino acids, which activate the Rag guanosine triphosphatases (GTPases), promote mTOR complex 1 (mTORC1) translocation to the lysosomal surface [10]. Upon amino acid treatment, vacuolar-type H^+^-adenosine triphosphatase (ATPase) (V-ATPase), a large multi-subunit enzyme, becomes associated with mTOR to enhance mTORC1 translocation to the lysosomal surface [10]. In addition, mTOR, which is a master growth regulator, promotes aerobic glycolysis and stimulates the expression of pyruvate kinase isozyme M2 (PKM2), a key glycolytic enzyme [11], indicating that proteins associated with the mTOR complex regulate nutrient-sensitive aerobic glycolysis in cancer cells. However, whether V-ATPase, which interacts with mTORC1, regulates glycolysis in ECCs is unclear.

Vacuolar ATPase plays an important role in pH homeostasis by regulating intensive respiratory CO_2_ and lactic acid production, which is required for cancer cell growth [12]. Vacuolar ATPase is organized by the V0 and V1 domains. The V0 domain, which comprises five different subunits, is involved in proton translocation [12]. On the other hand, the V1 domain, which comprises eight different subunits, is responsible for ATP hydrolysis [12]. Of all these subunits, subunit C of the V0 domain (ATP6V0C) is highly expressed in invasive pancreatic cancers [13]. Consistent with this result, fibroblast cells overexpressing ATP6V0C exhibit high invasiveness along with matrix metalloproteinase 2 (MMP-2) secretion [14]. Moreover, invasive ECCs heavily depend on glucose metabolism for energy supply [15]. However, how ATP6V0C regulates aerobic metabolism in ECCs is not fully understood.

In this study, we investigated the effect of ATP6V0C depletion on cell survival, migration, invasion, and aerobic glycolysis in ECCs. We also analyzed how ATP6V0C regulates aerobic glycolysis in ECCs.

## 2. Materials and Methods

### 2.1. Cell Culture and Transfection

Both TE8 and TE1 cells (RIKEN, Saitama, Japan) were cultured in RPMI-1640 Medium (Welgene, Inc., Gyeongsangbuk-do, Korea) containing 10% fetal bovine serum (Invitrogen, Carlsbad, CA, USA). The SH-SY5Y (ATCC, CRL-2266); HeLa (ATCC, CCL-2); SK-MEL-1 (ATCC, HTB-67); and U2OS cells (ATCC, HTB-96) were cultured in DMEM (Welgene, Inc., Gyeongsangbuk-do, Korea) containing 10% fetal bovine serum (Invitrogen, Carlsbad, CA, USA). Immortalized human esophageal normal fibroblasts (ENFs) were obtained from Seok-Hyung Kim (Department of Pathology, Samsung Medical Center, Sungkyunkwan University School of Medicine, Korea), a specialized gastrointestinal pathologist. He grossly examined esophageal tumor tissue and obtained samples of distal normal tissue [16,17]. The ENFs were cultured in DMEM/Ham’s F-12 (Welgene, Inc., Gyeongsangbuk-do, Korea) containing 10% fetal bovine serum (Invitrogen, Carlsbad, CA, USA). To suppress ATP6V0C expression, TE8, TE1, SH-SY5Y, HeLa, SK-MEL-1, and U2OS cells were transfected with a non-silencing control or ATP6V0C small interfering ribonucleic acids (siRNAs) using G-fectin (GP-2000) against ATP6V0C. The ENFs were transfected with a non-silencing control or ATP6V0C small interfering ribonucleic acids (siRNAs) using Lipofectamine RNAiMAX (Invitrogen, Carlsbad, CA, USA). The siRNA oligonucleotide sequence was as follows: si-ATP6V0C 5′-GCCTATGGCACAGCCAAGAGCGGTA-3′. A non-silencing control siRNA (Qiagen, Germany) was also used. The TE8, TE1, SH-SY5Y, HeLa, SK-MEL-1, U2OS cells, and ENFs were harvested 72 h after transfection.

### 2.2. RNA Purification and qRT-PCR

The RNA purification and quantitative reverse transcriptase polymerase chain reaction (qRT-PCR) were performed, as described previously [18]. Table S1 describes the primers used for qRT-PCR.

### 2.3. Cell Viability Assay

Aliquots of the TE8, TE1, SH-SY5Y, HeLa, SK-MEL-1, and U2OS cell suspensions were mixed with trypan blue dye and were left for 5 min at room temperature. Then, the TE8, TE1, SH-SY5Y, HeLa, SK-MEL-1, and U2OS cells were counted using a hemocytometer and the percentage of live cells was determined.

### 2.4. Western Blotting

Western blotting was performed, as described previously [19], using antibodies from the following sources: ATP6V0C (cat. NBP1-31492) from Novus Biologicals, Centennial, CO, USA. The PKM2 (cat. 4053), p-PKM2 (cat. 3827), p-Jun N-terminal kinase (p-JNK) (Thr183/Tyr185; cat. 9251), extracellular signal-regulated kinase (ERK) (cat. 9102), p-ERK (cat. 9101), p-paxillin (Tyr 118; cat. 2541), cleaved-caspase3 (cat. 9664), poly–adenosine diphosphate (ADP) ribose polymerase (PARP) (cat. 9542), activated protein kinase B (AKT) (cat. 9272), p-AKT (cat. 9271), 5′ adenosine monophosphate-activated protein kinase (AMPK) (cat. 2532), p-AMPK (cat. 2535), mTOR (cat. 2972), p-mTOR (cat. 2974), ribosomal protein S6 kinase 1 (S6K1) (cat. 9202), and p-S6K1 (cat. 9205) from Cell Signaling Technology, Inc., Danvers, MA, USA. c-Jun N-terminal kinase (c-JNK) (cat. sc6254), cyclin E (cat. sc25303), cyclin-dependent kinase 2 (cdk2) (cat. sc163), focal adhesion kinase (FAK) (cat. sc558), p-FAK (Tyr 397; cat. sc1688), b-cell lymphoma 2 (bcl-2) (cat. sc7381), Calnexin (cat. sc46669), Lamin B (cat. sc6217) and actin (cat. sc1616) were obtained from Santa Cruz Biotechnology, Inc., Santa Cruz, CA, USA. Paxillin (cat. BD610619) and lysosome-associated membrane protein 1 (Lamp-1) (cat. 555798) were obtained from BD Biosciences, Franklin Lakes, NJ, USA. Cyclooxygenase (Cox-1) (cat. 459600) was obtained from Invitrogen Corp., Carlsbad, California, USA.

### 2.5. Migration and Invasion Assays

An in vitro Matrigel (Becton Dickinson and Company, Franklin Lakes, NJ, USA) invasion assay was conducted using 8.0 µm (pore-size of inserts) Costar transwell chambers (Costar Inc., San Francisco, CA, USA). The transwell filters were coated with Matrigel (1 mg/mL). The TE8 cells were transfected with non-silencing (NS) or ATP6V0C siRNA for 72 h, and TE8 cells were then seeded again onto Matrigel at the same density of 5 × 10^4^ cells/well and cultured for 18 h. After incubation, the filters were removed from the chambers, the TE8 cells that had invaded the Matrigel were fixed and were stained with hematoxylin and eosin (H&E), and the number of TE8 cells attached to the filters was counted under a light microscope. A migration assay was conducted in a similar manner as the invasion assay, except that the filters were not coated with Matrigel. All assays were repeated a minimum of two times.

### 2.6. Immunoprecipitation

Precleared TE8 cell lysates were incubated with protein G Sepharose beads and primary antibodies or immunoglobulin G (IgG; control) overnight at 4 °C. The tubes were then centrifuged, and the supernatant was removed. The lysates were rinsed three times in PBS containing protease inhibitor. The proteins were eluted from the beads by adding 5× sample loading buffer. After centrifugation, Western blots were performed on the samples to analyze the precipitation of proteins.

### 2.7. GST Pull-Down Assay

Purified GST-PKM2 fusion protein and GST control (20 μg) were separately mixed with 20 μL of pre-equilibrated GST-agarose (GE Healthcare, Chicago, IL, USA) in sodium chloride tris-ethylenediaminetetraacetic acid (EDTA) (STE) buffer. The mixtures were incubated overnight at 4 °C with agitation and then washed thrice with STE buffer before elution with 50 mM reduced glutathione (in 50 mM Tris·HCl, pH 8.0). Protein samples from total cell extracts, immunoprecipitation (IP), GST pull-down, and chemical cross-linking were separated by 10% sodium dodecyl sulfate polyacrylamide gel electrophoresis (SDS-PAGE), followed by Western blotting with antibodies, as indicated.

### 2.8. Immunocytochemistry

The TE8 cells were blocked with bovine serum albumin (BSA) for 1 h. Then, the TE8 cells were immunostained using antibodies against ATP6V0C (cat. NBP1-31492), PKM2 (cat. 4053), Calnexin (cat. sc46669), Lamin B (cat. sc6217), Cox-1 (cat. 459600), and p-paxillin (Cell Signaling Technology). Next, cells were treated with an anti-rabbit fluorescein isothiocyanate (FITC)-conjugated secondary antibody (Invitrogen), an anti-mouse rhodamine-conjugated secondary antibody (Santa Cruz Biotechnology), and an anti-goat rhodamine-conjugated secondary antibody (Invitrogen) or Alexa 488 phalloidin (Invitrogen) containing 1 mg/mL Hoechst (Invitrogen); then, they were mounted with anti-fading mounting medium (Dako North America, Inc., Carpinteria, CA, USA). The TE8 cells were then visualized, and images were collected using fluorescence microscopy (Axiovert, Zeiss, Germany).

### 2.9. Lactate and ATP Assay

Lactate levels and ATP production in TE8 cell lysates were measured using a colorimetric L-Lactate Assay kit (Abcam, Cambridge, UK) and an EnzyLight^TM^ ADP/ATP Ratio Assay kit (BioAssay Systems, Hayward, CA, USA), respectively, according to the manufacturers’ instructions. The total number of cells was used for normalization.

### 2.10. Glucose Uptake Assay and Flow Cytometry

The TE8 cells were plated in 6 well plates at a density of 2 × 10^5^ cells/well for 24 h. Then, the TE8 cells were transfected with NS or ATP6V0C siRNA using G-fectin (GP-2000) under high- or low-glucose conditions. For the glucose uptake assay, all of the culture medium was removed from each well and replaced with 2 mL of the culture medium in the absence or presence of 10 μM fluorescent 2-deoxy-2-((7-nitro-2,1,3-benzoxadiazol-4-yl) amino)-D-glucose (2-NBDG). Then, the TE8 cells were incubated for 1 h at 37 °C with 5% CO_2_ before flow cytometry analysis. After removal of the incubation medium, the TE8 cells were washed twice with ice-cold PBS. This PBS (500 μL) was subsequently used to resuspend the cells. Then, 1 μg/mL of propidium iodide (PI) was added, and the TE8 cells were maintained at 4 °C for flow cytometry analysis using the BD FACS Canto II system (BD Biosciences, San Jose, CA, USA).

### 2.11. Annexin V Staining

The ENFs were washed twice with ice-cold PBS and then resuspended in 1× binding buffer to a concentration of 10^6^ cells/mL. One hundred microliters of resuspended cells were transferred to a 5 mL tube, and 5 μL of FITC Annexin V and 5 μL PI were added. The cells were gently vortexed and incubated for 15 min at room temperature (25 °C) in the dark. Four hundred microliters of 1× binding buffer was then added to each tube, and flow cytometric analysis performed within 1 h.

### 2.12. Measurement of Extracellular Acidification Rate and Oxygen Consumption Rate

The extracellular acidification rate (ECAR) and the oxygen consumption rate (OCR) of cultured TE8 cells were measured using a Seahorse XF24 extracellular flux analyzer according to the manufacturer’s instructions. Briefly, 25 mM glucose was added to stimulate cellular extracellular acidification and oxygen consumption, and 1 mM oligomycin (inhibitor of ATP synthase) was added to measure the glycolytic ability or ATP-linked OCR; 300 nM carbonyl cyanide *p*-(trifluromethoxy) phenylhydrazone (FCCP) (uncoupler of oxidative phosphorylation in mitochondria) was added to determine mitochondria respiration capacity. Finally, 100 nM rotenone (inhibitor of mitochondrial respiratory chain complex I) was added to inhibit mitochondrial electron transport chain (ETC).

### 2.13. Subcellular Fractionation

To prepare cytosolic and nuclear lysates, TE8 cells were lysed in buffer A (10 mM 4-(2-hydroxyethyl)-1-piperazineethanesulfonic acid (HEPES), pH 7.9; 10 mM KCl; 0.1 mM EDTA; 1 mM dithiothreitol (DTT); 0.5% NP-40; and protease inhibitors) and incubated on ice for 15 min. The cytosolic fractions were harvested by centrifugation at 13,000 rpm for 2 min at 4 °C. Next, the nuclear pellets were washed with ice-cold PBS to avoid the mixing of cytosolic proteins, lysed in buffer B (20 mM HEPES, pH 7.9; 400 mM NaCl; 1 mM EDTA; 1 mM DTT; and protease inhibitors), and incubated on ice for 10 min. The nuclear fractions were harvested by centrifugation at 13,000 rpm for 10 min at 4 °C.

### 2.14. Chromatin Immunoprecipitation Assay

The TE8 cells were seeded onto 10 cm dishes in RPMI 1640 Medium 1 day before treatment. The TE8 cells were incubated in RPMI 1640 Medium containing 25 mM glucose for 6 h, as previously described [19]. A chromatin immunoprecipitation (ChIP) assay was conducted using PKM2 antibodies (cat. H00005315-M01A; Abnova, Taiwan). Table S1 describes the primers used for qRT-PCR.

### 2.15. Statistical Analysis

The viability, migration, and invasion assay results were analyzed using one-way analysis of variance (ANOVA), two-way ANOVA, or Student’s *t*-tests. Differences among individual group means were analyzed by ANOVA or two-tailed *t*-tests. All assay data were presented as mean ± standard error of the mean (SEM). A two-sided significance level of 0.05 was used for all statistical analyses.

## 3. Results

### 3.1. ATP6V0C Depletion Inhibits ECC Proliferation

**Figure 1:**
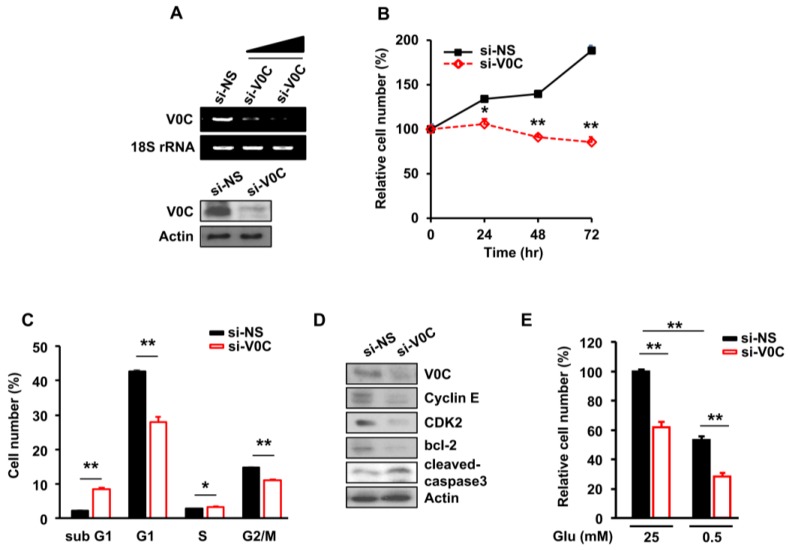
The vacuolar H^+^-adenosine triphosphatase (ATPase) subunit V0C (ATP6V0C) is crucial for esophageal cancer cell (ECC) proliferation in the presence of glucose. The TE8 cells were transfected with non-silencing siRNA (NS) or si-V1E1 for 72 h. (**A**) Whole-cell lysates from ATP6V0C-depleted TE8 cells were evaluated for ATP6V0C expression using RT-PCR or Western blotting. (**B**) Cell viability was analyzed using a trypan blue assay. (**C**) Cell cycle analysis was performed using flow cytometry. (**D**) TE8 cell lysates were analyzed with the indicated antibodies using Western blotting. (**E**) With high- (25 mM) or low- (0.5 mM) glucose conditions, the cell number was analyzed using a trypan blue assay. (**B**–**E**) Each data point is the average of three independent measurements. Values are mean ± SEM. (In (**B**), ANOVA; in (**C**,**E**), Student’s *t*-test; * *p* < 0.05; ** *p* < 0.01).

The ATP6V0C is essential for maintaining the pH gradient between the cytoplasmic and lysosomal compartments [20]. The intracellular pH gradient regulates cell growth through pH-sensing proteins, such as G-actin and F-actin [21]. However, whether ATP6V0C affects ECC growth is unclear. To investigate this, we performed ATP6V0C depletion using siRNAs in TE8 cells, which have the highest colony-forming ability among the TE1, TE2, and TE8 cell lines [22] (Figure 1A).

Cell proliferation was markedly reduced (Figure 1B) and the portion of sub-G1 cells related to apoptosis increased in ATP6V0C-depleted TE8 cells (Figure 1C). In addition, we performed ATP6V0C depletion using siRNAs in TE1 cells, another esophageal cancer cell line. The ATP6V0C depletion also led to the reduction in TE1 cell proliferation (Figure S1), suggesting that the effect of ATP6V0C depletion was not cell-line specific.

To determine whether the reduction of ATP6V0C levels is toxic to ENFs, we used siRNAs to deplete ATP6V0C and evaluate the effect on the cells. The proliferation of ENFs was unaffected by ATP6V0C depletion when compared to cells treated with NS (Figure S2).

In exploring whether ATP6V0C regulates the expression of cell-cycle regulators and cell survival-related proteins in TE8 cells, we found that reducing levels of ATP6V0C decreased the expression of cyclin E, cyclin-dependent kinase 2 (cdk2), and b-cell lymphoma (bcl-2), indicating G1 phase arrest (Figure 1D). In addition, levels of cleaved caspase 3, an apoptosis marker, substantially were increased by ATP6V0C depletion (Figure 1D). However, in ENFs, the levels of cleaved caspase 3, poly (ADP-ribose) polymerase (PARP), apoptosis-related proteins, and bcl-2, a regulator of cell survival, were unchanged by ATP6V0C depletion (Figure S2). Moreover, apoptosis in ENFs was not significantly reduced by the depletion of ATP6V0C, as revealed by Annexin V staining (Figure S2).

We further investigated whether ATP6V0C depletion also affects the proliferation of other cancer cell lines. The ATP6V0C depletion led to a reduction in proliferation of neuroblastoma SH-SY5SY, cervix adenocarcinoma HeLa, melanoma SK-MEL-1, and osteosarcoma U2OS cells (Figure S3). Interestingly, the expression levels of ATP6V0C varied depending on the cancer cell line (Figure S3), suggesting a potential role for ATP6V0C in various cancers.

Given that most cancer cells use glucose as the main carbon source for anabolic growth [23], we examined whether ECC survival depends on glucose availability. The ATP6V0C depletion resulted in a 40% decrease in the proliferation of TE8 cells compared to cells treated with non-silencing siRNA (NS) under high-glucose condition (Figure 1E). Similarly, after ATP6V0C depletion, we found a 50% decrease in the TE8 cell number under low-glucose conditions (Figure 1E).

These results suggested that ECC viability is sensitive to glucose availability and ATP6V0C depletion results in a decrease in the survival of ECCs; this was not observed with ENFs.

### 3.2. ATP6V0C Depletion Attenuates *Cell Adhesion*, Migration, and Invasion In ECCs

**Figure 2:**
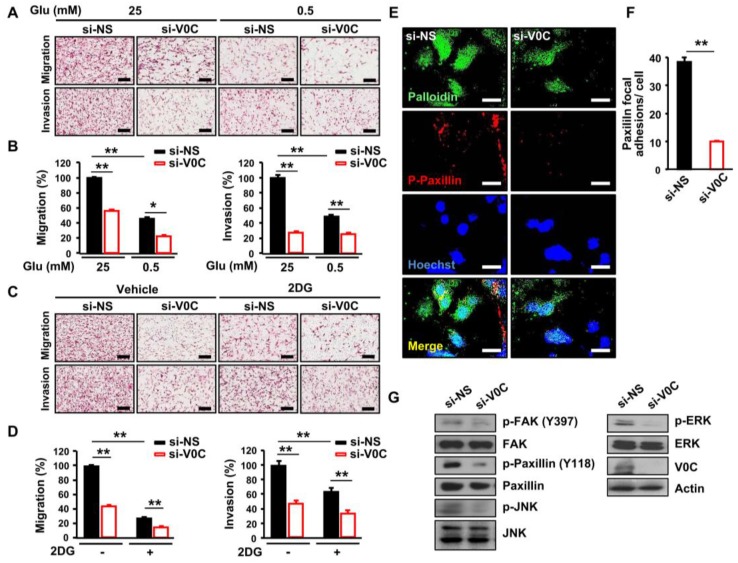
ATP6V0C depletion attenuates migration, invasion, and formation of focal adhesions in ECCs. (**A**–**D**) TE8 cells were transfected with NS or si-V0C. (**A**,**B**) Cell migration and invasion assays were performed after transfection under high- (25 mM) or low- (0.5 mM) glucose conditions. (**C**,**D**) Images showing migration and invasion assays in 2-deoxy-D-glucose (2DG) (5 mM) treated TE8 cells. (**A**–**D**) Cells were fixed and stained with H&E, and the slides of filters of migrated or invaded cells were scanned using an Aperio scanner (original magnification 100×). Scale bars = 400 μm. (**E**) TE8 cells were immunostained with p-paxillin antibody and rhodamine-labeled secondary antibody, Hoechst, and Alexa 488 phalloidin, and then viewed by fluorescence microscopy. Scale bars = 50 μm. (**F**) The numbers of paxillin focal adhesions per cell were determined. (**G**) TE8 cell lysates were analyzed by immunoblotting with the indicated antibodies. Assays and blots are representative of two independent experiments. Values are mean ± SEM. (ANOVA; * *p* < 0.05, ** *p* < 0.01).

Maintenance of intracellular pH homeostasis is essential for cancer cell motility leading to metastasis, and an acidic environment is enhanced by proton-sensing proteins [24]. Therefore, we examined whether depletion of ATP6V0C, which is a pH regulator, affects TE8 cell motility and invasiveness under high- or low-glucose conditions. The ATP6V0C depletion inhibited migration and invasion of TE8 cells (Figure 2A,B). In addition, low-glucose condition and ATP6V0C depletion decreased further both migration and invasion of TE8 cells by 50% (Figure 2A,B).

Inhibition of glycolysis using 2-deoxy-D-glucose (2DG) suppresses the metastatic phenotype in colorectal cancer [25]. Therefore, we examined whether 2DG can attenuate further the metastatic potential of TE8 cells with ATP6V0C depletion. We found that combining ATP6V0C depletion and 2DG resulted in greater inhibition of migration and invasion of TE8 cells compared to 2DG treatment alone, suggesting that both ATP6V0C and glucose are critical for migration and invasion of ECCs (Figure 2C,D). In addition, the adhesive properties of gap junctions are necessary for migration and invasion of cancer cells [26]. Therefore, we examined the effect of ATP6V0C depletion on TE8 cell adhesion. We analyzed the phosphorylation levels of FAK and paxillin, a multidomain adaptor between the plasma membrane and the actin cytoskeleton [27]. The ATP6V0C depletion led to pronounced inhibition of TE8 cell adhesion, as revealed by decreased levels of phosphorylated paxillin (Figure 2E,F). We also assessed the phosphorylation status of signaling components that mediate motility and adhesion in ATP6V0C-depleted TE8 cells and found that ATP6V0C depletion inhibits phosphorylation of FAK, ERK, and c-JNK in ECCs (Figure 2G). These results indicate that ATP6V0C induces phosphorylation of signaling components, including ERK, JNK, and FAK, during ECC adhesion and movement.

### 3.3. ATP6V0C Enhances Aerobic Glycolysis in ECCs

**Figure 3:**
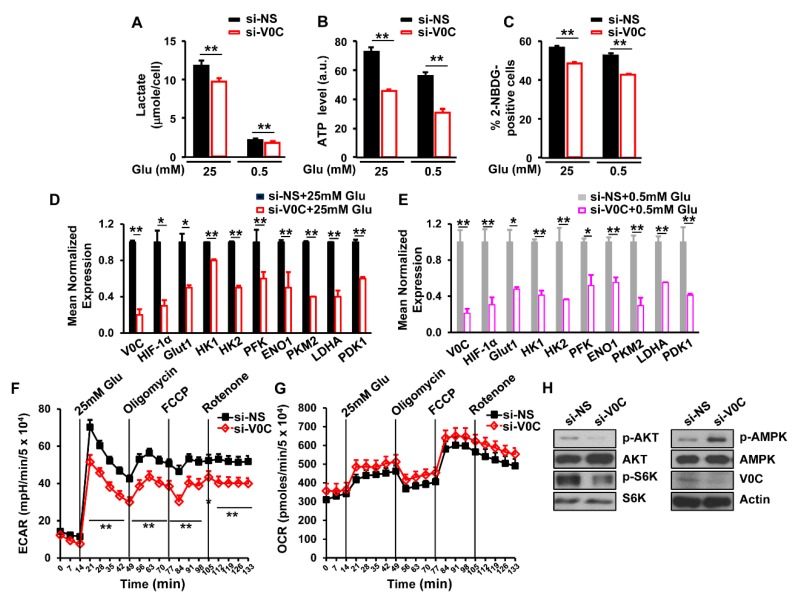
ATP6V0C depletion decreases glycolysis in ECCs. (**A**,**B**) Effects of ATP6V0C depletion on lactate or ATP levels in TE8 cells. (**C**) Effect of ATP6V0C depletion on glucose uptake. TE8 cells were transfected with NS or si-V0C for 72 h and then were treated with 10 μM 2-NBDG, a fluorescent indicator for direct glucose uptake. Glucose uptake was analyzed by flow cytometry. (**D**,**E**) Gene expression related to glycolytic enzymes or glucose transport in ATP6V0C-depleted TE8 cells by qRT-PCR. (**F**) Extracellular acidification rate (ECAR) or (**G**) oxygen consumption rate (OCR) in ATP6V0C-depleted TE8 cells after treatment of inhibitors. TE8 cells were starved of glucose for 1 h prior to initiation of the experiment. Inhibitors were added at indicated time points: 25 mM glucose, 1 mM oligomycin, 300 nM FCCP, and 100 nM rotenone. Experiments were conducted in the absence of sodium pyruvate. (**H**) Expression of indicated proteins in TE8 cells treated with NS or si-V0C. (**A**–**E**) Each data point is the average of three independent measurements. Values are mean ± SEM. (In (**A**–**E**), Student’s *t*-test; in (**F**,**G**), ANOVA; * *p* < 0.05; ** *p* < 0.01).

Glucose metabolism facilitates cancer cell proliferation by increasing the synthesis of ATP and lactate, which are the final products of glycolysis [5]. Therefore, we assessed the effects of ATP6V0C depletion on ATP and lactate levels in TE8 cells under high- or low-glucose conditions. We observed that ATP6V0C depletion significantly reduced ATP and lactate production compared to cells treated with NS under both high- and low-glucose conditions (Figure 3A,B). In addition, ATP6V0C depletion resulted in a significant decrease in glucose uptake under high- and low-glucose conditions (Figure 3C).

Glucose uptake enhances the expression of hypoxia inducible factor 1 (HIF-1) target genes, such as glucose transporter 1 (*Glut1*), hexokinase II (*HK2*), and lactate dehydrogenase A (*LDHA*), in aerobic glycolysis [28]. To determine whether ATP6V0C affects the expression of glycolytic genes, we measured messenger RNA (mRNA) levels of HIF-1 target genes. We found that ATP6V0C depletion resulted in decreased expression of glycolytic genes such as *Glut1, HIF-1, HK2*, phosphofructokinase-1 (*PFK1*), enolase 1 (*ENO1*), *PKM2*, *LDHA*, and pyruvate dehydrogenase lipoamide kinase isozyme 1 (*PDK1*), under both high- and low-glucose conditions (Figure 3D,E), suggesting that ATP6V0C positively regulates the expression of HIF 1-dependent glycolytic genes.

The ECAR and OCR qualitatively reflect the rates of lactic acid production through glycolysis and mitochondrial respiration, respectively [29]. Analysis of whether ATP6V0C depletion affects the ECAR and OCR demonstrated that ATP6V0C knockdown did not have a significant effect on the basal level of ECAR in the absence of glucose (Figure 3F). However, upon glucose stimulation, depletion of ATP6V0C resulted in a significant reduction of ECAR (Figure 3F). When oligomycin, which inhibits ATP synthase in mitochondria, was added, ECAR was further decreased by ATP6V0C depletion (Figure 3F). Similarly, ATP6V0C knockdown led to lower ECAR compared with the NS control after treatment with FCCP which induces proton leak, or rotenone, a complex I inhibitor to shut down electron transport chain (Figure 3F). However, there was no significant difference in OCR between NS and *ATP6V0C* knockdown cells after treatment with mitochondrial inhibitors, oligomycin, FCCP, or rotenone (Figure 3G). Thus, these results suggest that ATP6V0C is critical for glycolysis rather than mitochondrial respiration in ECCs in the presence of glucose.

Both AKT and S6K enhance energy metabolism and proliferation, while AMPK is activated under metabolic stress by interfering with the catabolic generation of ATP [30,31]. In this study, we examined the effects of ATP6V0C depletion on phosphorylation levels of AKT, S6K1, and AMPK. The ATP6V0C depletion decreased AKT and S6K1 phosphorylation but increased AMPK phosphorylation, while the total levels of AKT, S6K1, and AMPK remained unchanged (Figure 3H), indicating that ATP6V0C enhances signaling related to energy metabolism for ECC growth. Taken together, the results of this study suggest that ATP6V0C increases aerobic glycolysis and maintains ECC proliferation under both high- and low-glucose conditions.

### 3.4. ATP6V0C Interacts with PKM2 and Increases Its Phosphorylation at Tyr^105^

**Figure 4:**
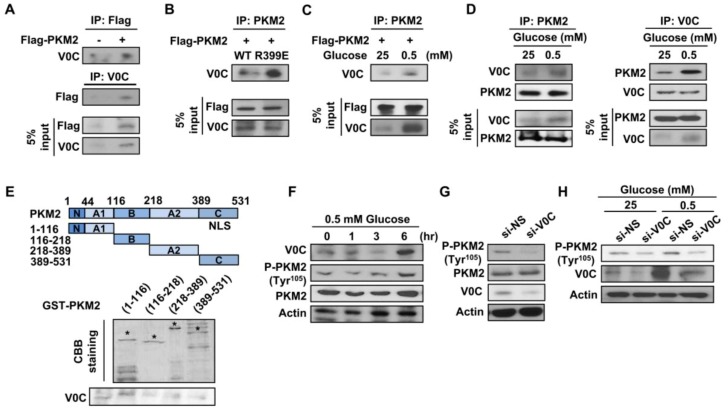
ATP6V0C interacts with PKM2 and increases PKM2 phosphorylation at Tyr^105^. (**A**,**B**) Reciprocal IP were performed in TE8 cells transfected with expression vectors such as Flag-PKM2 or the Flag-PKM2 mutant R399E. (**C**) Under high- or low-glucose conditions, co-immunoprecipitation assays were performed with an anti-Flag antibody in TE8 cells transfected with Flag-PKM2. (**D**) IP assays were performed with anti-ATP6V0C or anti-PKM2 in TE8 cells, followed by Western blotting. (**E**) GST pull-down assays were performed with GST alone or GST-PKM2 fusion protein expressed in bacterial cells. (**F**) TE8 cells were treated with 0.5 mM glucose during the indicating time point and, then, were analyzed for protein expression by Western blotting. (**G**) Western blotting of indicated proteins in TE8 cell lysates treated with NS or si-V0C. (**H**) TE8 cells were transfected with indicated siRNA under high- (25 mM) or low- (0.5 mM) glucose conditions, followed by Western blotting.

The PKM2, a key regulator of glucose metabolism, increases lactate production but decreases oxygen consumption [32], leading to cancer cell growth and motility. The PKM2 interacts with HIF-1, leading to an increase in aerobic glycolysis; therefore, we examined whether ATP6V0C interacts with PKM2. The results revealed that endogenous ATP6V0C is co-immunoprecipitated with overexpressed Flag-PKM2 in TE8 cells (Figure 4A). The PKM2 mutant R399E, common in cancer cells, disrupts tetramer formation of PKM2, thereby producing dimers and decreasing its pyruvate kinase activity, eventually resulting in increased cancer cell proliferation [32,33]. Therefore, we analyzed whether ATP6V0C is associated with R399E. The ATP6V0C was associated more strongly with the Flag-PKM2 mutant R399E compared to Flag-PKM2 WT (Figure 4B), suggesting that ATP6V0C interacts with dimeric PKM2.

Next, we determined whether such an association is influenced by glucose availability. Interestingly, compared to high-glucose conditions, endogenous ATP6V0C strongly interacted with Flag-PKM2 (Figure 4C) and interacted more with endogenous PKM2 in TE8 cells (Figure 4D) under low-glucose condition.

The PKM2 domain containing amino acids 1–116 includes catalytic active sites, such as tyrosine residue 105 (Tyr^105^) and tyrosine residue 148 (Tyr^148^) [34], which are significant in cancer cell metabolism and growth. To determine the physical interaction between PKM2 and ATP6V0C, we conducted cell-free GST pull-down assays using GST-PKM2. The results demonstrated that the N-terminal domain of PKM2 containing amino acids 1–116 interacts strongly with ATP6V0C, whereas other PKM2 portions containing amino acids 116–218, 218–389, or 389–531 are not associated with ATP6V0C (Figure 4E). These results also indicate that ATP6V0C preferably binds to the N-terminal domain of PKM2 containing amino acids 1–116.

Tyrosine phosphorylation (Tyr^105^) of PKM2 increases PKM2 activity, leading to enhanced aerobic glycolysis [35]. Therefore, we investigated whether ATP6V0C expression affects PKM2 phosphorylation under high- or low-glucose conditions. We found that ATP6V0C expression and PKM2 phosphorylation increased in a time-dependent manner (0–6 h) under low-glucose conditions (Figure 4F). In addition, ATP6V0C depletion decreased PKM2 phosphorylation under both high- and low-glucose conditions (Figure 4G,H). These results suggest that ATP6V0C interacts with the catalytic domain of PKM2 and enhances PKM2 phosphorylation to facilitate glycolysis in ECCs.

### 3.5. ATP6V0C Induces Nuclear Translocation of PKM2 and Increases Glycolytic Gene Expression

**Figure 5:**
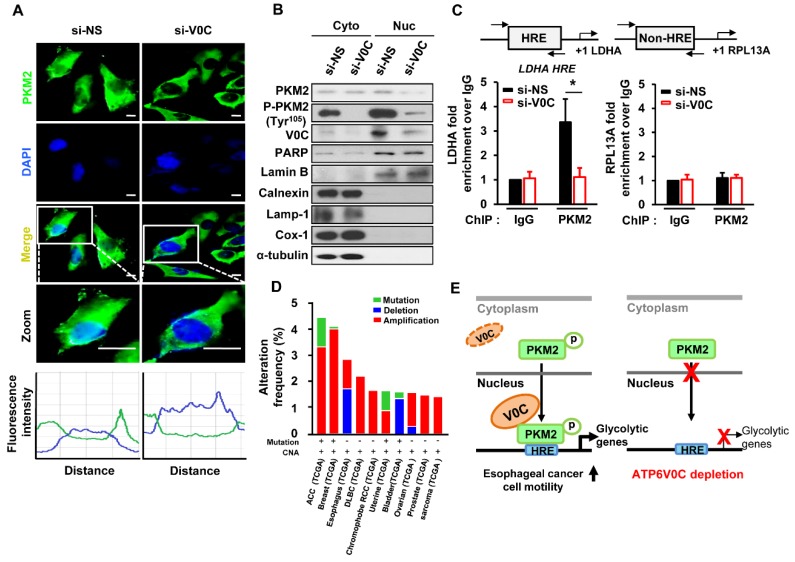
ATP6V0C increases expression of glycolytic genes by inducing nuclear translocation of PKM2. (**A**) TE8 cells were immunostained with PKM2 antibody (PKM2, green). The nucleus is marked with DAPI (blue). Merged images (Merge) are shown. Fluorescenceintensity was shown in linear histogram. The intensity was divided into two groups: PKM2 fluorescence intensity is plotted on the green peak, and DAPI intensity is plotted on the blue peak. The line profiles were placed across cells, and the simultaneously occurring intensity peaks were evaluated. The line profiles of PKM2 and DAPI signals were measured by ZEN 2011 software (Carl Zeiss). Scale bars = 10 μm. (**B**) Nuclear and cytosolic lysates were prepared from ATP6V0C-depleted TE8 cells, followed by immunoblotting. (**C**) Recruitment of PKM2 by ATP6V0C at the HRE site of the indicated promoter. TE8 cells were transfected with NS or si-V0C and subjected to ChIP assays. Each data point is the average of three independent measurements. Values are mean ± SEM. (Student’s *t*-test; * *p* < 0.05). (**D**) Frequency of copy number alterations and mutations of ATP6V0C across several tumor types. (**E**) Proposed model of how ATP6V0C mediates metabolic programming in ECCs. ATP6V0C associates with PKM2 to enhance its nuclear localization and increases PKM2 phosphorylation, inducing transcriptional expression of glycolytic genes and thus facilitating glycolysis and motility in ECCs.

An increase in PKM2 dimer formation and a subsequent decrease in pyruvate kinase activity induce nuclear localization of PKM2, which eventually serves as a coactivator for glycolytic gene transcription [36]. Therefore, we analyzed whether ATP6V0C depletion changes nuclear localization of PKM2. The ATP6V0C depletion inhibited nuclear translocation of PKM2 and decreased phosphorylated PKM2, indicating that ATP6V0C is critical for nuclear translocation of PKM2 (Figure 5A) and its phosphorylation at Tyr^105^. Interestingly, ATP6V0C depletion prevented nuclear accumulation of PKM2 and decreased phosphorylated PKM2 at Tyr^105^, leading to decreased expression of glycolytic enzymes (Figure 5B). We further determined the subcellular localization of ATP6V0C using immunofluorescence staining. Our analysis revealed that ATP6V0C was mainly localized in the nucleus (Figure S4). Intriguingly, ATP6V0C was partially co-localized with Calnexin, Lamp-1 or Cox-1 (Figure S4), indicating its potential role in other organelles such as the ER, lysosome, or mitochondria in ECCs.

We investigated using the ChIP assay whether ATP6V0C stimulates PKM2 recruitment to hypoxia response element (HRE) sites of *LDHA*. Generating lactate ATP6V0C depletion significantly decreased PKM2 accumulation at the *LDHA* HRE site but not at *RPL13A* (the non-HIF-1 target gene), in TE8 cells (Figure 5C), suggesting that ATP6V0C enhances PKM2 association to the *LDHA* HRE site.

To determine the clinical relevance of ATP6V0C expression in esophageal cancer, we retrieved whole exome sequencing and mRNA expression data from 10,967 patients with cancers from The Cancer Genome Atlas (TCGA) database. Genetic changes in ATP6V0C, particularly its deletion or amplification, were frequently found in esophageal cancer, underscoring the significance of ATP6V0C in tumors (Figure 5D). The results shown here are based upon data generated by the TCGA Research Network [37].

Together, these results suggest that ATP6V0C induces the nuclear translocation of PKM2, leading to enhanced transcriptional activation of glycolytic genes, and eventually resulting in stimulation of glycolysis in ECCs (Figure 3, Figure 4, and Figure 5E).

## 4. Discussion

The vacuolar-ATPase is composed of a cytosolic V1 domain for ATP hydrolysis and a transmembrane V0 domain, which drives proton translocation across the membrane [12]. The vacuolar H+-ATPase is a proton pump that induces the acidification of intracellular vesicles, such as endosomes and lysosomes [38]. Activated proton pumps regulate intracellular pH by modulating proton translocation into intracellular organelles [38]. However, whether ATP6V0C regulates motility and glucose metabolism of highly invasive ESCC cells is unclear. In this study, we showed that ATP6V0C depletion resulted in a decreased proliferation accompanied by increased apoptosis of ECCs but had no effect on the viability of ENFs. Our results agree with other studies which have shown that viability of normal human dermal fibroblasts is unaffected by ATP6V0C depletion [39,40]. Similarly, recent studies have demonstrated that ATP6V1C1 knockdown does not affect the growth of mouse embryonic fibroblasts, C3H10T1/2, while reducing the proliferation of breast cancer cells such as MCF-7, MDA-MB-231, and MDA-MB-435S [41]. In addition, treatment with bafilomycin A1, a V-ATPase inhibitor exhibited no effect on the viability of normal human gingival fibroblasts, although greatly reducing the viability of Ewing sarcoma cells such as A-673 and SK-N-MC [42]. In addition, ATP6V0C depletion also led to a reduction in the proliferation of various cancer cells, such as SH-SY5SY, HeLa, SK-MEL-1, and U2OS cells. Thus, we speculate that when ATP6V0C is targeted using a knockdown, cancer cells are heavily affected as glycolysis is their predominant energy source. The ATP6V0C is critical for such metabolism, whereas normal cells are less affected, due to the low levels of dependence on glycolysis, and rely more on mitochondrial respiration for energy.

Our analysis of TCGA data further confirmed that genomic alteration of ATP6V0C is frequently observed in ESCC, suggesting its clinical significance in esophageal squamous cancer. Consistent with our findings, bafilomycin, concanamycin, and archazolid, which decrease ATP6V0C activity, inhibit the growth and proliferation of breast, prostate, and leukemia cancer cells respectively [43,44,45], suggesting a role for ATP6V0C in the survival of breast, prostate, and leukemia cancer cells. In addition, overexpression of ATP6V0C induces the secretion of neurotransmitters such as acetylcholine, serotonin, and dopamine [46]. These studies and our results suggest that ATP6V0C plays multiple roles in various cancer and neuronal cells.

Our study further demonstrated that ATP6V0C positively regulates glucose metabolism, which produces main energy sources for esophageal cancer cells. Lowering levels of ATP6V0C resulted in reduced glucose uptake and decreased lactate and ATP production under both high- and low-glucose conditions, leading to downregulation of glycolysis. These results suggested that ATP6V0C might prevent cell death by enhancing glycolysis under low-glucose conditions.

We assessed further how ATP6V0C regulates aerobic glycolysis to maintain cell proliferation under low-glucose conditions. In this study, ATP6V0C expression was increased further under low-glucose conditions. Consistent with this, ATP6V0C depletion prevented low-glucose-induced expression of glycolytic enzymes. In addition, ATP6V0C depletion decreased the ECAR, an indicator of aerobic glycolysis, whereas there was no significant difference in OCR between the NS and *ATP6V0C* knockdown cells after treatment with mitochondrial inhibitors, suggesting that ATP6V0C is crucial for glycolysis in ECCs.

The ATP6V0C depletion inhibits the expression of glycolytic enzymes and decreases glycolysis, the first step in glucose oxidation. Studies have revealed that glucose deprivation contributes to mutations in the KRAS pathway [47] and induces the Warburg effect [48]. Moreover, aerobic glycolysis generating ATP directly associates with ATP-hydrolyzing proton pumps by interacting V-ATPase with aldolase, as a glucose sensor [49]. Therefore, these and our own results suggest that ATP6V0C enhances glycolytic metabolism, depending on glucose availability.

During glycolysis, pyruvate kinase (PK), which catalyzes the transfer of a phosphate from phosphoenolpyruvate to pyruvate, contributes to active glucose metabolism in cancer cells [32]. While PKL, PKR, and PKM1 form stable tetramers (active forms of PK), PKM2 exists as both a dimer and a tetramer [50], and its activity is dynamically regulated by switching between the less active PKM2 dimer and the highly active PKM2 tetramer [51]. The PKM2 tetramer favors ATP production through the tricarboxylic acid cycle, while the PKM2 dimer leads to increased levels of intermediates (e.g., glycerate 3-P, glyceraldehyde 3-P, and glucose 6-P) in glycolysis [51], suggesting that the PKM2 dimer is necessary for high rates of cell proliferation. Our results revealed that endogenous ATP6V0C interacts with PKM2 in TE8 cells. The PKM2 binds to HRE, leading to expression of PKM2 target genes and increase of aerobic glycolysis.

The molecular mechanisms underlying the interaction of ATP6V0C with PKM2 in TE8 cells are unclear. Our findings revealed that ATP6V0C associates preferentially with the PKM2 mutant R399E compared to WT PKM2. The PKM2 mutant R399E exists as a dimer and appears to be catalytically inactive for the conversion of phosphoenolpyruvic acid (PEP) to pyruvate [33]. The inactive PKM2 dramatically facilitates the proliferation and growth of tumor cells from the SW480 cell line [52]. In addition, PKM2 overexpression is significantly associated with the advanced tumor grade of ESCC [53]. Therefore, it is possible that ATP6V0C interacts with the PKM2 mutant R399E or PKM2, driving glucose metabolism and leading to high-grade esophageal cancer. Further studies are required to determine whether the PKM2 mutant R399E exists in esophageal cancer and its relevance to altered glucose metabolism.

The assembly of the vacuolar H+-ATPase complex occurs when high levels of glucose are available, whereas glucose depletion induces V-ATPase disassembly leading to the inhibition of V-ATPase activity [54,55]. Interestingly, the interaction between ATP6V0C and PKM2 was stronger under low- compared to high-glucose conditions. Moreover, studies have shown that PKM2 activity is stimulated by succinyl-5-aminoimidazole-4-carboxamide-1-ribose-5-phosphate (SAICAR), one of the metabolites abundant in actively proliferating cells, and that the interaction between PKM2 and SAICAR promotes cancer cell survival under glucose starvation [56]. Based on our results and other studies, the positive role of ATP6V0C in glycolysis is predominant when glucose availability is low along with a decrease of V-ATPase activity. Thus, future studies should explore whether the interaction between ATP6V0C and PKM2 upon energy stress leads to an increase in aerobic glycolysis for ECC survival.

The ATP6V0C and PKM2 phosphorylation increased under low-glucose conditions. The ATP6V0C depletion led to a decrease in PKM2 phosphorylation at Tyr^105^. In addition, ATP6V0C depletion resulted in inhibition of PKM2 nuclear translocation. Relevant to this, previous studies have reported that erythroblastic oncogene B, a receptor tyrosine kinase 2, induces PKM2 phosphorylation at Tyr^105^ by increasing nuclear localization of Yes-associated protein, and enhances cancer cell-like properties [57]. The fibroblast growth factor receptor 1 has an intracellular domain with tyrosine kinase activity and directly induces PKM2 phosphorylation at Tyr^105^. In addition, the PKM2 Y105F mutation leads to a reduction in lactate production and an increase in oxidative phosphorylation [35], pointing to the significance of phosphorylated PKM2 at Tyr^105^. Our results suggest that ATP6V0C indirectly affects phosphorylation of PKM2 during glycolysis. Considering both that an increase in PKM2 phosphorylation at Tyr^105^ is positively associated with nuclear localization of PKM2 [58] and our data, ATP6V0C is likely to induce nuclear translocation of PKM2 by enhancing PKM2 phosphorylation at Tyr^105^, although the kinase responsible has not yet been identified in esophageal cancer cells.

The ChIP analysis revealed that ATP6V0C depletion results in reduced recruitment of PKM2 to HRE sites, the gene promoter for transactivation of *LDHA*, which catalyzes the intra-conversion of pyruvate and lactate to enhance glycolysis.

In conclusion, glucose-sensitive ATP6V0C interacts with the catalytic domain of PKM2 containing Tyr^105^ and leads to nuclear translocation of PKM2 to *LDHA* HRE sites, enhancing transcriptional activation of glycolytic genes and, thus, inducing metabolic reprogramming and movement of ECCs. Therefore, targeting ATP6V0C could be an effective therapeutic approach for controlling deregulated glucose metabolism in ECCs.

## Supplementary Materials

The following are available online at https://www.mdpi.com/2073-4409/8/10/1137/s1, Figure S1: ATP6V0C is crucial for ECC proliferation. Figure S2: The effect of ATP6V0C siRNA on viability of esophageal normal fibroblasts (ENFs). Figure S3: ATP6V0C depletion attenuates proliferation of various cancer cell lines. Figure S4: The sub-cellular localization of ATP6V0C in TE8 cells. Table S1: Primers used for quantitative RT- PCR.
