# Supplementary material for: Vacuolar H+-ATPase Subunit V0C Regulates Aerobic Glycolysis of Esophageal Cancer Cells via PKM2 Signaling
Source: Cells. 2019 Sep 24;8(10):1137. doi: 10.3390/cells8101137 (PMC6830105; doi:10.3390/cells8101137)
Supplement: Supplementary file 1 [file cells-08-01137-s001.pdf]

Supplementary Figures and Table

Vacuolar H^+-ATPase subunit V0C regulates aerobic glycolysis of esophageal cancer cells via PKM2 signaling

Sung Wook Son[1, 2], Gia Cac Chau[1, 2], Seong-Tae Kim[1], and Sung Hee Um[1, 2, 3 *]

[1] Department of Molecular Cell Biology, Samsung Biomedical Research Institute, Sungkyunkwan University School of Medicine, Suwon, Gyeonggi-do, 16419, Korea; ssw4069@gmail.com (S.S.); chaugiacac@gmail.com (G.C.C.); stkim@skku.edu (S.K.)

[2] Department of Health Sciences and Technology, Samsung Advanced Institute for Health Sciences and Technology, Samsung Medical Center, Sungkyunkwan University, Seoul, 06351, Korea

[3] Biomedical Institute Convergence at Sungkyunkwan University, Suwon, Gyeonggi-do, 16419, Korea

* Correspondence: shum@skku.edu; Tel: +82-31-299-6123; Fax: +82-31-299-6109

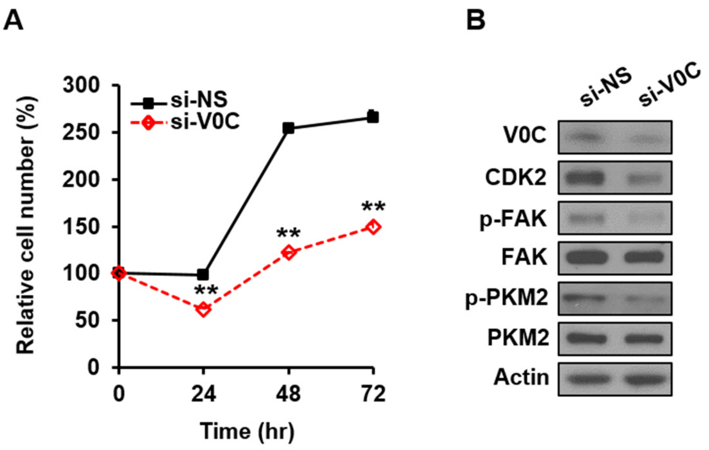

Supplementary Figure S1. ATP6V0C is crucial for ECC proliferation. TE1 cells were transfected with non-silencing siRNA (NS) or si-V0C for 72 h. (**A**) Cell viability was analyzed using a trypan blue assay. Each data point is the average of three independent measurements. (**B**) Western blot showing the expression of the indicated proteins in ATP6V0C-depleted TE1 cells. Values are mean ± SEM. (Student's *t*-test; $^{**}P < 0.01$).

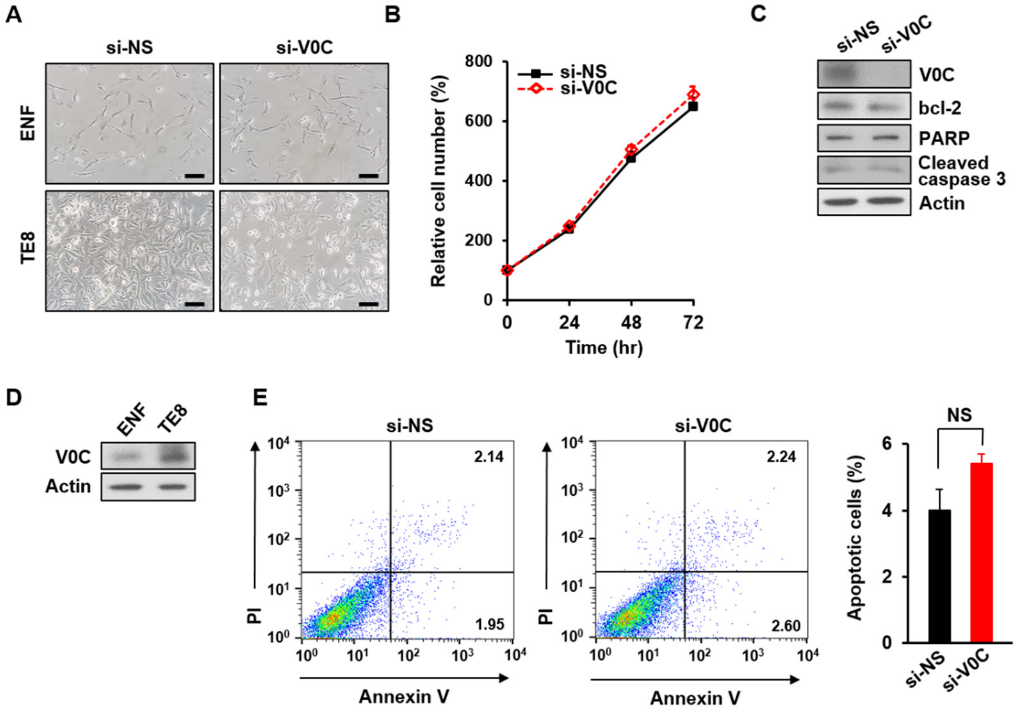

Supplementary Figure S2. The effect of ATP6V0C siRNA on the viability of Esophageal Normal Fibroblasts (ENFs). Cells were transfected with 50 nM of non-silencing siRNA (NS) or si-V0C for 72 h. (**A**) Representative images of ENFs and esophageal cancer cell line (TE8). Scale bars = 100 μm. (**B**) Cell viability was analyzed using a trypan blue assay. (**C**) Cell lysates were analyzed by immunoblotting using the indicated antibodies. (**D**) ENF lysates were analyzed by immunoblotting with the indicated antibodies. (**E**) Representative flow cytometry plots of Annexin V staining in ENFs (percentage of apoptosis cells stained with Annexin V). Values are mean ± SEM. (Student's *t*-test; non-significant (NS)).

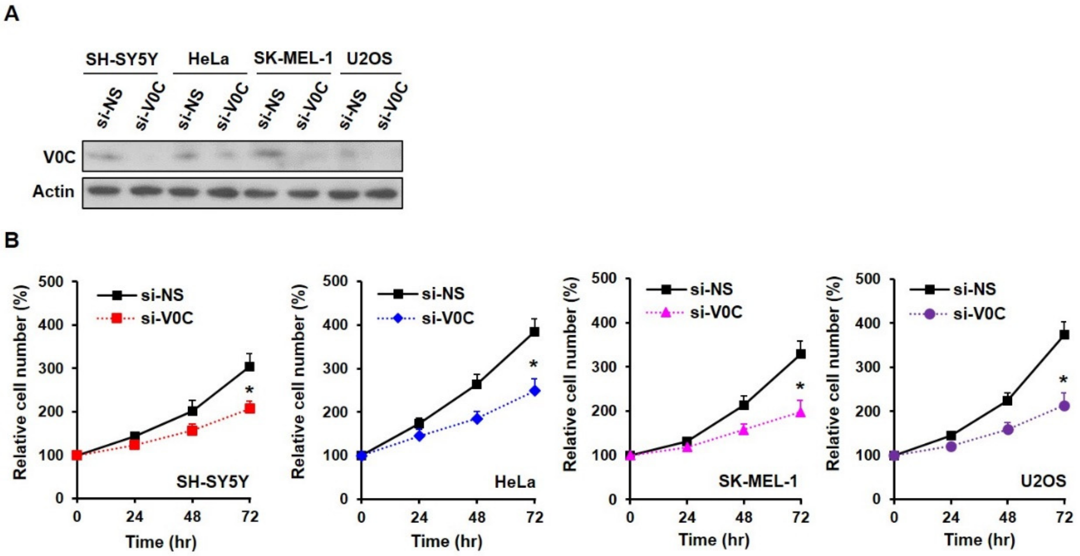

Supplementary Figure S3. ATP6V0C depletion attenuates proliferation of various cancer cell lines. SH-SY5Y, HeLa, SK-MEL-1, and U2OS cells were transfected with non-silencing siRNA (NS) or si-V0C for 72 h. (**A**) ATP6V0C and Actin expression were analyzed using Western blotting. (**B**) Cell viability was analyzed using a trypan blue assay. Each data point is the average of three independent measurements. Values are mean ± SEM. (Student's *t*-test; *$P < 0.05$).

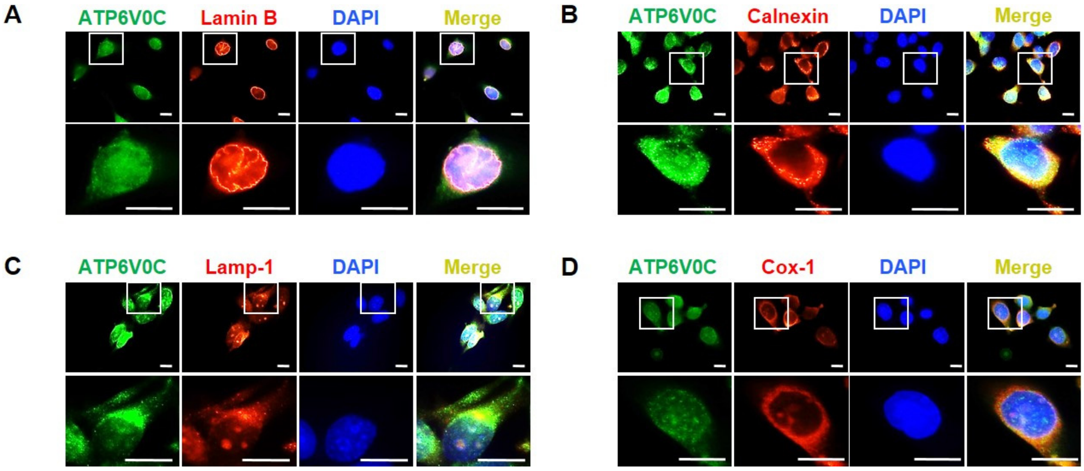

Supplementary Figure S4. The sub-cellular localization of ATP6V0C in TE8 cells. Co-immunostaining analysis was performed using specific antibodies for (**A**) ATP6V0C (green), Lamin B (red), (**B**) Calnexin (red), (**C**) Lamp-1 (red) (**D**) Cox-1 (red) in TE8 cells. Cell nuclei were counterstained with DAPI (blue). Merged images (Merge) are shown. Scale bars = 20 μm.

Supplementary Table S1: Primers used for quantitative RT-PCR

Primer name	sequence
**hV-ATPase V0C-F**	5'-AGC AGA TCA TGA AGT CCA TC-3'
**hV-ATPase V0C-R**	5'-GAC GAT GAG ACC GTA GAG G -3'
**hHIF-1a-F**	5'-CGT TCC TTC GAT CAG TTG TC-3'
**hHIF-1a-R**	5'-TCA GTG GTG GCA GTG GTA GT-3'
**hGlut1-F**	5'-CGG GCC AAG AGT GTG CTA AA-3'
**hGlut1-R**	5'-TGA CGA TAC CGG AGC CAA TG-3'
**hHK1-F**	5'-TGG AGT CCG AGG TTT ATG-3'
**hHK1-R**	5'-TTT GGA TTG TTG GCA AGG-3'
**hHK2-F**	5'-CAA AGT GAC AGT GGG TGT GG-3'
**hHK2-R**	5'-GCC AGG TCC TTC ACT GTC TC-3'
**hPFK-F**	5'-CAT GAC CCA TGA AGA GCA CC-3'
**hPFK-R**	5'-CCA ACT CGA ACC ACA GCC CTG-3'
**hENO1-F**	5'-CGT ACC GCT TCC TTA GAA C-3'
**hENO1-R**	5'-CAA TGA CTT GGG CCA ATT AC-3'
**hPKM2-F**	5'-GCC TGC TGT GTC GGA GAA G-3'
**hPKM2-R**	5'-CTC TCC CAG GAC CTT CCT AA-3'
**hLDHA-F**	5'-ATC TTG ACC TAC GTG GCT TGG A-3'
**hLDHA-R**	5'-CCA TAC AGG CAC ACT GGA ATC TC-3'
**hPDK1-F**	5'-ACC AGG ACA GCC AAT ACA AG -3'
**hPDK1-R**	5'-CCT CGG TCA CTC ATC TTC AC -3'
**hLDHA-HRE-F**	5'-TTG GAG GGC AGC ACC TTA CTT AGA-3'
**hLDHA-HRE-R**	5'-GCC TTA AGT GGA ACA GCT ATG CTG AC-3'
**hRPL13A–non-HRE-F**	5'-GAG GCG AGG GTG ATA GAG-3'
**hRPL13A–non-HRE-R**	5'-ACA CAC AAG GGT CCA ATT C-3'
**h18s rRNA F**	5'-CGG CGA CGA CCC ATT CGA AC-3'
**h18s rRNA R**	5'-GAA TCG AAC CCT GAT TCC CCG TC-3'
